# Wireless Local Area Network Link Sharing in Unmanned Surface Vehicle Control Scenarios

**DOI:** 10.3390/s26020751

**Published:** 2026-01-22

**Authors:** Krzysztof Gierłowski, Michał Hoeft, Andrzej Bęben, Maciej Sosnowski

**Affiliations:** 1 Faculty of Electronics, Telecommunications and Informatics, Gdańsk University of Technology, Gabriela Narutowicza 11/12, 80-233 Gdańsk, Poland; michal.hoeft@pg.edu.pl; 2 Institute of Telecommunications and Cybersecurity, Warsaw University of Technology, Nowowiejska 15/19, 00-665 Warsaw, Poland; andrzej.beben@pw.edu.pl (A.B.); maciej.sosnowski@pw.edu.pl (M.S.)

**Keywords:** USV communication, WLAN, traffic prioritization, laboratory experiments, field tests

## Abstract

The popularity of unmanned vehicles in numerous areas of employment, combined with the diversity and continuing evolution of their payloads, make the communication solutions utilized by such vehicles the element of a particular importance. In our previous publication, we confirmed a general applicability of wireless local area network (WLAN) technologies as solutions suitable to provide a control loop communication of unmanned surface vehicles (USVs). At the same time, our research indicated that WLAN technologies provide communication resources in excess of what is required for the above task. In this paper, we aim to verify if a WLAN-based USV communication solution can be reliably utilized for both time-sensitive control loop and high-throughput payload communication simultaneously, which could provide significant advantages during USV construction and operation. For this purpose, we analyzed traffic parameters of popular USV payloads, designed a test system to monitor the impact of such traffic sharing a WLAN link with a USV control loop communication and conducted laboratory and field experiments. As initial results indicated the significant impact of payload traffic on the quality of control communication, we have also proposed a method of employing Commercial Off The Shelf (COTS) hardware for this purpose, in a manner which allows the above-mentioned link sharing to operate reliably in changing real-world conditions. The subsequent verification, first in the laboratory and then during a real-world USV field deployment, confirmed the effectiveness of the proposed method.

## 1. Introduction

The usefulness of unmanned vehicles (UVs) in different areas, starting with entertainment, through commercial use-cases and ending with emergency and military applications, is evident [1], while unmanned air vehicles (UAVs) receive the most publicity due to their popularity in the consumer market, other types of UVs are also frequently deployed, especially to provide commercial services. In the case of unmanned surface vehicles (USVs), which are our focus in this paper, their employment scenarios include such areas as bathymetry [2], biological research [3,4], environment monitoring [5,6], maritime inspection/survey [7] and a wide range of military applications [8], including reconnaissance, logistics, mine sweeping and strike missions.

The complexity of the performed tasks and the unpredictability of the operating environment make reliable communications with a human operator a necessity, despite the growing popularity of autonomous control functions, which allows USVs to perform basic tasks on their own. Well-established solutions, such as dedicated, direct narrowband communication links in 433 MHz, 868 MHz or 2.4/5 GHz bands, are sufficient for Visual Line of Sight (VLOS) control, but the ability to carry control protocols (such as Micro Air Vehicle Link protocol, abbreviated MAVLink and being a widely recognized standard for exchanging messages used to monitor and control unmanned vehicles) [9] encapsulated in Internet Protocol datagrams creates a possibility of employing a wide range of wireless technologies that are popular in computer communications in USV operations [10].

As USVs are often employed in areas lacking the coverage of modern public communication infrastructure (such as LTE/5G networks)—for example due to operating at sea at a considerable distance from an on-shore network infrastructure or in areas where the infrastructure has been destroyed/cannot be considered reliable (disaster response and military scenarios)—the interest in employing popular wireless local area network (WLAN) technologies as a dedicated, private USV communication solution has arisen. Our previous research indicates that WLAN technologies such as Wi-Fi (based on IEEE 802.11 standard [11]) and TDMA-based WLAN solutions, such as proprietary NV2 [12], are capable of supplying communication capabilities that are sufficient for direct, real-time USV control and telemetry [10]. Their low cost, availability of devices and ease of deployment due to operation in unlicensed bands make them highly attractive solutions. Moreover, WLAN technologies provide broadband communication capabilities, so a communication link used for USV control and telemetry retains a large amount of free resources—possibly enough to carry payload-related communications, making it an all-in-one solution for USV mission support.

However, it is well known that transmitting different types of traffic flows over a shared link can lead to their adverse interaction and bring quality of service (QoS) parameters (such as transmission delay) for these flows below acceptable levels. The risk is especially high for wireless communication links, where the amount of available resources changes depending on current propagation conditions.

In this paper, we focus on Wi-Fi technology to propose a method of its employment that is capable of providing communication capabilities sufficient to support an all-in-one scenario, where both USV control/telemetry and payload-related communications share the same wireless link. The choice of Wi-Fi has been made based on the popular availability of compatible devices designed for varied environments (including industrial), ongoing interest from USV users [13,14,15] and our previous research, which indicates its functional advantages (e.g., low communication delay) but cautions against resource pool changes due to changing propagation conditions [10].

For this purpose, in Section 2, we provide a short reminder of the methods of employing Wi-Fi connectivity in USV control loop, complete with laboratory and field-experiment results confirming their viability. In Section 3, an assessment of network traffic requirements and the characteristics of popular USV payloads (such as sonar, LiDAR, etc.) is presented. This allows us to present, in Section 4, the design and results of laboratory experiments verifying the possibility and methods of obtaining the coexistence of USV control-loop and payload-related network traffic. Section 5 describes real-world deployment and measurement results, confirming the results in Section 4 for a series of field-experiments. Section 6 concludes the paper and highlights its most important contributions, complete with a set of experimentally verified configuration best practices aimed to facilitate the use of Wi-Fi technology for a shared control/payload USV communication.

## 2. Applicability of Wi-Fi Communication for Surface-Drone Control

**Figure 1:**
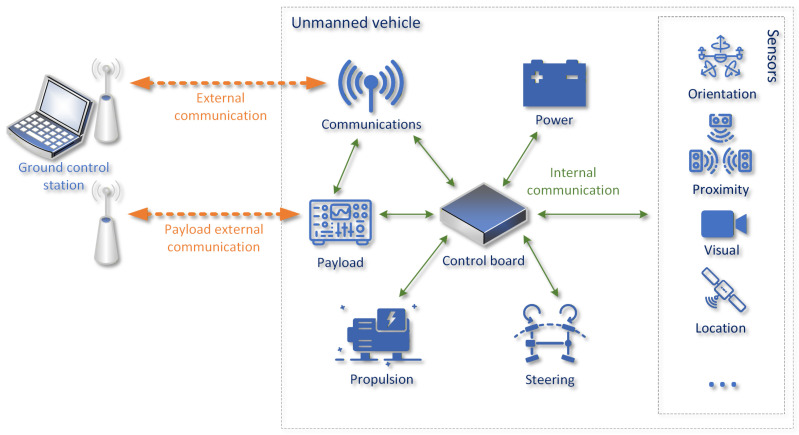
General architecture of USV control system (extended from [10]).

Modern USVs consist of a number of subsystems that are responsible for different tasks necessary for an USV to operate. Most often, these subsystems are integrated using a centralized controlling element, to which they are connected using internal communication interfaces (see Figure 1). These interfaces are usually standardized wired connections, but their particular type depends on the communication requirements of a particular subsystem (e.g., a high-resolution visual sensor will utilize a different communication interface than a propulsion control module). Such a solution ensures the sufficient quality and reliability of internal communications, despite significant differences in requirements.

**Figure 2:**
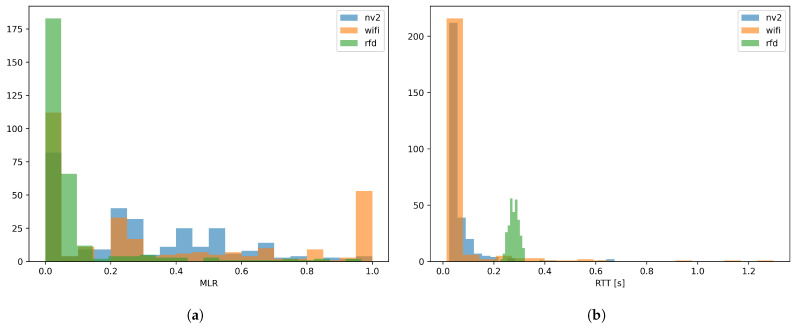
MAVLink protocol (**a**) Message Loss Ratio and (**b**) Round Trip Time for a Wi-Fi USV communication link (real-world experiments presented in [10]).

Apart from internal communications, a USV most often needs to maintain contact with its operator (a control loop) by means of external communication link. Additionally, a payload element (which is not a subsystem required for the basic operation of an USV) can introduce its own external communications solution instead of sharing the link with the USV control loop, to minimize the probability of negatively impacting the operation of this time-critical element. Such approach is popular due to the fact that payload functionality and communication requirements may vary significantly, and thus are difficult to take into account when designing an integrated communication solution. Our previous experiments [10] confirmed the general usefulness of WLAN technologies for the purpose of maintaining a USV control loop, indicating the high reliability of TDMA-based solutions such as NV2 [12] (a contention-free, proprietary WLAN technology) (Figure 2a) and very short communication delays offered by contention-based Wi-Fi (Figure 2b) wireless links.

The detailed results of real-world experiments are presented in [10], but for the purpose of the current paper, can be summarized by the plots included in Figure 2, which show the values of two key quality metrics of MAVLink protocol communication:

- Message Loss Ratio (MLR)—the ratio of messages which were not responded to by an unmanned vehicle to a total number of messages sent to it by a ground station. While control protocols such as MAVLink are designed to tolerate a certain level of message loss [16], excessive losses can lead to the erratic behavior of unmanned vehicle while under remote operator control, or the inability of a ground station to update the configuration and/or mission plan of such a vehicle.
- Round Trip Time (RTT)—the time required for a ground station to receive a response for a control message sent to an unmanned vehicle. The value of this parameter directly influences the responsiveness of the control loop, and its minimization is especially important in case of remotely controlled vehicles requiring quick and frequent operator input.

The plots show the frequency of values of the above metrics measured in 15 s intervals for communication links created using different WLAN technologies (Wi-Fi, NV2) and an RFD 868 MHz wireless link [17]—a proprietary, narrowband technology specifically designed for use in unmanned vehicle control applications. The results were obtained for a USV operating at a range between 50 m and 2300 m from the ground station. The operating environment included a set of conditions representative for the standard commercial mission for such a USV type: the USV remaining in VLOS conditions and within the sector of ground station antenna most of the time, but with relatively short (up to 1 min) periods when the USV operated outside of the sector or when VLOS was obscured by shore vegetation. The full environment description and discussion of obtained results are available in [10], but a cursory glance at the figure shows us, that while the WLAN technologies suffer a higher (but still acceptable) message loss compared to the dedicated RFD radio link, their RTT results are visibly better—the RTT values for Wi-Fi (<150 ms) allow it to be placed into group of solutions suitable for controlling all maneuvers with fixed-wing unmanned air vehicles without a noticeable impact on the quality of experience for an experienced pilot [18], significantly exceeding general requirements for fixed-wing UAV remote control (300 ms) [19].

Such a result significantly surpasses the requirements of USVs, which are characterized by a relatively slow response to control inputs, and thus can tolerate much larger delays—in specific cases, even as large as 2 s, without a substantial decrease in operator control efficiency [20]. The observed parameters are within acceptable levels for USV control loop [18,19], and relatively higher message loss in the case of Wi-Fi technology occurred only in during periods where direct line of sight between USV and ground station was obscured.

An observation of particular interest is that both broadband WLAN technologies retained an unused transmission capacity in range of 3–35 Mbps during the experiment, in contrast with the narrowband RFD solution, which retained about 150 kbps.

These results clearly indicate that, in contrast to narrowband solutions, it should be possible to utilize WLAN links to carry additional, payload-related traffic, making such a link a comprehensive communication solution for a mission-configured USV. At the same time, WLAN susceptibility to propagation environment changes (resulting in a significant degradation in the available data throughput) makes it necessary to ensure that the USV control loop will not be disrupted by payload traffic sharing such a link. For this purpose, in the following section, we start our analysis by reviewing the communication requirements of popular USV payloads.

## 3. USV Payload Communication Characteristics

Unmanned surface vehicles (USVs) rely on advanced perception systems to gather mission-specific data. These systems generate substantial volumes of data, imposing requirements on bandwidth and latency for data transfer between the USV and its remote control station [21]. Some of the most popular examples include the following:

1. RADAR (Radio Detection and Ranging) [22] provides medium- and long-range object detection and tracking. RADAR ensures robust, all-weather performance, enhancing navigation safety and situational awareness.
2. LiDAR (Light Detection and Ranging) [23,24] delivers high-resolution, near-range environmental mapping through real-time laser scanning. LiDAR generates detailed 3D point clouds but is sensitive to adverse conditions such as fog, rain, and sea spray.
3. High-resolution 360° cameras and visual sensors [25] offer comprehensive azimuthal coverage for object classification, situational awareness and surveillance.
4. SONAR (Sound Navigation and Ranging) serves as the primary technology for underwater detection, ranging, and imaging.

In the following sections, we present typical data volumes generated by these sensors, along with the corresponding transmission requirements and the standards used.

### 3.1. RADAR Systems

USVs use marine X-band radar systems for navigation, collision avoidance, and situational awareness. These radars operate at the 9.3–9.5 GHz band and provide ranges of a few to tens of kilometers, typically consuming a power of 30 to 40 W. The exemplary radars are the Furuno DRS4WI and Simrad HALO20+ FMCW systems.

The performance of these radars depends on several key parameters. Range resolution improves with increased bit rates, enabling better target recognition at varying distances. Similarly, azimuth resolution, which determines angular precision, increases data volume as it becomes finer. The update rate typically varies between 1 and 10 Hz, depending on the operational mode—higher rates are used for collision avoidance, while lower rates suffice for surveillance. Radar data can be processed at various levels, including raw I/Q samples, detected targets, or fully processed track information. For example, raw radar data streams range from 10 to 500 Mbps and are rarely sent off the vehicle. Instead, processed target data is transmitted for remote monitoring, typically requiring a bandwidth of between 100 Kbps and 5 Mbps.

### 3.2. LiDAR Systems

LiDAR is a key technology for USV, providing precise three-dimensional perception of the surrounding environment. Unlike radar, which uses radio waves, LiDAR relies on laser pulses to measure distances and create dense point clouds. This capability makes it indispensable for tasks such as collision avoidance, high-definition mapping, and environmental monitoring, where centimeter-level accuracy is required. A LiDAR sensor emits rapid laser pulses and measures the time it takes for each pulse to return after reflecting off an object. By combining these measurements with angular information, the system constructs a detailed 3D representation of the scene. Each point in this cloud typically contains X, Y, and Z coordinates, an intensity value, and a timestamp, resulting in approximately 18–24 bytes per point. Modern LiDARs generate hundreds of thousands to millions of points per second, resulting in a substantial data volume. For example, a high-end mechanical LiDAR can produce around 2 million points per second, which translates to an uncompressed data rate of nearly 384 Mbps. Even after compression, the required bit rate remains high—typically between 100 and 200 Mbps. Mid-range mechanical LiDARs, commonly used in USV applications, generate approximately 600,000 points per second, requiring 115 Mbps of uncompressed data and 30–50 Mbps of compressed data. Solid-state LiDARs, which are more compact and robust, produce lower data rates (around 58 Mbps uncompressed), while single-line LiDARs used for basic ranging operate at just a few Mbps.

### 3.3. High-Resolution 360° Cameras and Visual Sensors

Visual sensors provide rich contextual information for object classification, situational awareness, and operator surveillance. Notably, 360° panoramic camera systems provide complete azimuthal coverage, which is essential for autonomous navigation and surveillance. These systems may consist of a single panoramic camera or a multi-camera rig with 4–8 cameras, offering resolutions ranging from 1080p to 8K and frame rates of 15–30 frames per second (fps).

Stereo vision systems can provide depth perception, operating at 1080p–4K resolution and 30–60 frames per second, with depth ranges of up to 50 m. Thermal and infrared cameras are indispensable for night operations and low-visibility conditions, working in the 8–14 μm LWIR (Long-Wave Infrared) band. Monocular cameras provide basic obstacle detection and monitoring at lower complexity. Environmental challenges include lens contamination from salt spray, glare from sunlight, fog, and motion blur caused by wave-induced movement. Solutions involve optical cleaning systems, HDR imaging, thermal cameras, and image stabilization.

While video data rates can be substantial, reaching as high as 19.9 Gbps uncompressed for an 8K panoramic stream at 30 fps (which H.265/HEVC compression can reduce to 50–150 Mbps), typical high-end multi-camera rigs consume 40–50 Mbps after compression.

### 3.4. SONAR Systems

SONAR (Sound Navigation and Ranging) is the primary sensor for underwater perception on USVs. By emitting acoustic pulses and measuring the echoes, sonar enables bathymetric mapping, submerged hazard detection, and imaging below the waterline where cameras and LiDAR are ineffective due to water turbidity and light attenuation [26]. Operating at acoustic frequencies (tens to hundreds of kHz), sonar sensors have inherently lower data generation rates and higher latency compared to optical sensors due to the limited propagation speed of sound and narrow underwater bandwidth [27]. A single sonar ping’s round-trip travel takes 100–500 ms for ranges of tens to hundreds of meters, so overall data update rates are on the order of a few hertz. Raw sonar returns (e.g., acoustic intensity images or depth point clouds) are often processed on-board into concise measurements before transmission. Typical sonar data streams range from a few kilobits per second for simple depth finders up to a few hundred kilobits per second for high-resolution imaging sonars. These modest bandwidth requirements mean that even low-bandwidth links can support sonar data. For instance, advanced compression enables streaming of side-scan sonar imagery at under 2 kbps over acoustic modems [28].

Environmental factors, such as temperature, salinity, and ambient noise, primarily affect the sonar signal-to-noise ratio, range, and image quality, which in turn influence the amount of useful data gathered. However, they have little direct impact on the capacity of the USV–shore communication link. Overall, sonar sensors produce moderate-volume, delay-tolerant data that is well-suited to edge processing and opportunistic transmission in USV networks.

In conclusion, we point out that USVs can employ a wide variety of payload types including RADAR, LiDAR, vision cameras, and sonar. Each of them produces a different volume of data to be processed on-board or transmitted to the control station for processing, operator awareness, advanced analysis and archiving. The bandwidth requirements depend on operational needs, but in most cases, high-throughput traffic sources such as LiDAR or high-resolution vision systems will require a bit rate of up to 20–50 Mbps. Based on this analysis, we have decided to employ a 50 Mbps constant bitrate (CBR) traffic stream in the following practical experiments as an example of a high-throughput payload traffic. This choice is not an obvious one, because many USV payloads will generate variable bitrate (VBR) traffic streams (for example, due to employment of data compression). However, the use of predictable CBR traffic allows us to easily observe and analyze the impact of external influences on the system under test. As our main goal is to verify a proposed method of Wi-Fi link sharing, while our initial laboratory experiments (presented in Section 4.1) indicate that until the network becomes overloaded, the changes in QoS for control communication are minor, we decided to use a CBR payload stream to easily observe and analyze system operation in field conditions.

## 4. Laboratory Testbed Design and Experiment Scenarios

As shown in the preceding section, a transmission rate comparison indicates that standard WLAN technologies (such as IEEE 802.11n [29] and later) can theoretically provide communication resources sufficient for a wide range of popular USV payloads. However, in the case of practical deployments, the operation of such a contention-based access technology will be influenced by multiple factors. In this situation, to verify the practical applicability of the technology for the above-mentioned task, it has been decided to conduct a real-world deployment and measurements.

With field experiments being both time-consuming and simultaneously including a large number of factors in the assessment, they were preceded by laboratory experiments aimed to provide results obtained in a strictly controlled environment. Such results allowed us to both design the real-world experiments to specifically include scenarios important for communication performance in this particular use case and to easily interpret the obtained results.

**Figure 3:**
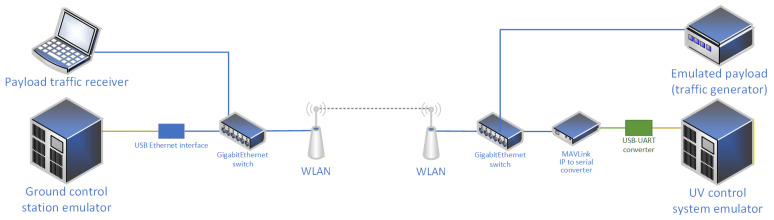
Architecture of a testbed system designed for both laboratory and field experiments.

In both laboratory and field experiments, we have employed a modification of a test setup designed for our previous research regarding the general applicability of WLAN technologies in USV control tasks [10]. The current version of the setup is shown in Figure 3 and includes an emulated on-board USV control system and its corresponding ground control station (GCS), communicating using the MAVLink protocol. Because in the case for most USV implementations, the MAVLink protocol is available over a serial interface (widely used for internal USV communication), a MAVLink-over-Serial to MAVLink-over-IP (MoIP) converter has to be employed. It has been implemented using a single-board computer (Raspberry Pi 4B) with a USB-attached RS232 interface, running a well-known MAVProxy software release v1.8.74 [30] and configured to utilize UDP encapsulation. On the GCS side, MoIP is supported natively by the GCS software utilizing a USB-attached Gigabit Ethernet network interface.

To retain result continuity with our previous research [10], we employed a Metal 52ac (Mikrotik, Riga, Latvia) device with an omnidirectional 6 dBi antenna on the USV side, while on the GCS side, we used a mANT 15 (Mikrotik, Latvia) with an inbuilt 15 dBi sector (120 °H/10 °V) antenna as WLAN communication devices, providing ISO-OSI layer 2 connectivity between USV and GCS. The radio link was configured to use IEEE 802.11ac [31] communication in 5 GHz ISM band over a 20 MHz channel. Transmission power was configured to conform with Polish regulatory requirements and thus did not exceed 20 dBm EIRP. All other configuration parameters relevant to WLAN communication were left at their default values. The above radio communication devices were connected to a USV MoIP converter and GCS network interface using Gigabit Ethernet switches completing a communication circuit between the USV control system and its GCS.

As our goal is to verify the possibility of sharing a wireless communication link between USV control and payload traffic, on the USV side, an IP traffic generator was been connected to the Gigabit Ethernet switch, while on the GCS side, a separate computer connected to the switch received the simulated payload traffic. It was verified that Gigabit Ethernet switches introduce negligible delay (commonly <20 μs [32])—the same for both control and payload traffic.

**Figure 4:**
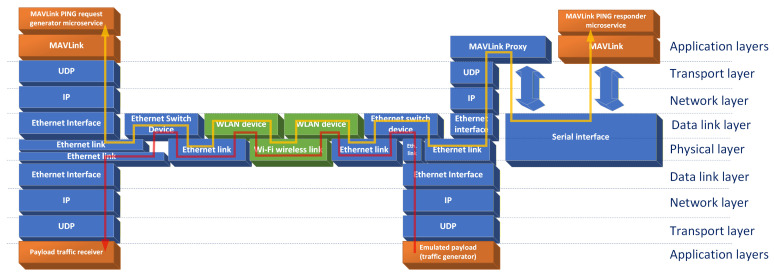
A network communication model of the employed USV communication test system with MoIP control traffic flow marked by a yellow arrow and payload traffic flow marked as a red arrow.

The network model for the test system is presented (in context of ISO-OSI model) in Figure 4. The GCS mechanisms are located on the left of the figure, and we can see that they have a native support for MAVLink protocol transmission over the IP/UDP protocol stack. To generate MAVLink messages simulating USV control traffic, a dedicated MAVLink microservice was implemented. It is capable of generating MAVLink PING request messages according to a traffic pattern described in experiment descriptions. An IP/UDP payload traffic receiver protocol stack is also depicted on the left of the figure, including an application capable of receiving such a traffic stream. Both GCS and payload traffic receiver are connected to an Ethernet switch, which allows IP/UDP datagrams to be delivered to a WLAN device.

The WLAN device, using only link-layer processing, forwards MAVLink messages (carried in IP/UDP datagrams) received via Ethernet connection over the wireless link to a similar device terminating its USV side. The operation of this pair of devices is of crucial importance to the task of providing a USV control link of acceptable parameters and sharing its resources between control and payload traffic in an acceptable manner.

On the USV side, the MAVLink traffic is carried by an Ethernet connection to an Ethernet switch, and then, by another Ethernet link, to a protocol converter based on an application-layer proxy software and utilizing IP/UDP stack on the network side, while being connected to an emulated USV control system by a direct serial connection. The serial connection does not use any network or transport layer protocols, directly passing binary data to a MAVLink PING responder microservice that we implemented. As our main focus in this paper is to propose and verify an effective way of sharing a wireless link between control and payload traffic, a constant bitrate IP/UDP payload traffic generator was implemented on the USV side and connected to the same Ethernet switch as the MAVLink protocol converter.

As can be seen in Figure 4, the delay-sensitive MoIP control traffic and the high-intensity payload traffic share a common transmission path including two Ethernet switching devices, two Ethernet links and a Wi-Fi wireless link between a pair of WLAN devices. As the used Ethernet devices and links employ Gigabit Ethernet technology, their impact on the obtained measurements should be minimal and unchanging.

In the described test system, we had the GCS sending MAVLink PING messages to the USV control system every 200 ms. As a MAVLink PING message contains a timestamp and requires the receiving device to immediately respond with a similar message to the sender, it allowed us to measure both the MAVLink Message Loss Ratio (MLR) and Round Trip Time (RTT) for a two-way communication of USV control loop. The message size was 112 bits, which resulted in a bidirectional MAVLink data stream of 1200 bps in each direction (see Table 1).

We need to emphasize that in the case of remotely controlled USVs, it is likely that the actual MAVLink traffic will diverge from our assumption described above. This is probable, especially if we consider that control inputs from the operator of the USV will generate additional messages to be transmitted, resulting in a VBR stream. However, for the purpose of measuring and analyzing the impact of a wireless link on the timing characteristics of MAVLink communication, we can consider such a traffic model for a MAVLink communication as appropriate. Moreover, the model has also been utilized in other publications [33,34] and employed in our previous paper, where we consulted with a manufacturer of the commercial USV (Hydrodron by Marine Technologies, Gdynia, Poland) [10] used in experiments.

The laboratory experiments have been conducted with GCS and USV antennas located in an RF-shielded enclosure [35], thus eliminating external radio signals, which could unpredictably impact the obtained results.

As the first experiment, we have considered a special case, where instead of a radio link between Gigabit Ethernet switches, we employ a cable connection offering effectively lossless communication with transmission speeds exceeding the requirements of the control traffic by a few orders of magnitude. The results were used as a baseline for comparison with radio-link scenarios.

**Figure 5:**
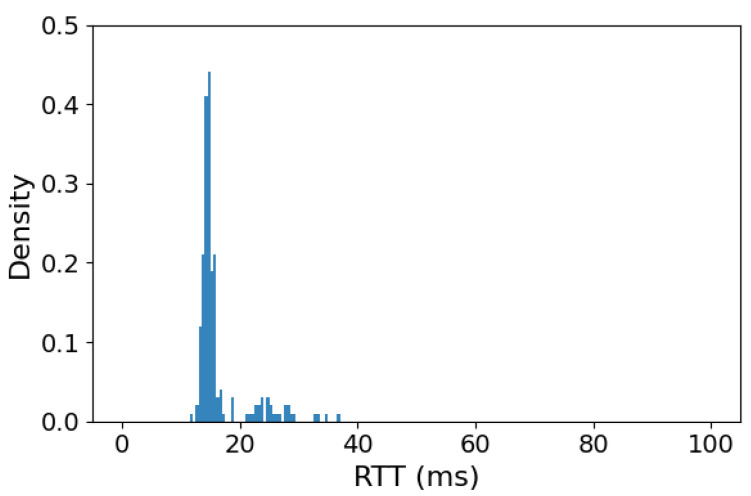
MAVLink over IP communication Round Trip Time histogram for test system using a cable Gigabit Ethernet connection between GCS and USV sides.

The obtained results (presented in Figure 5) confirm the correctness of test system operation and illustrate the most optimistic case that is realistically possible for such a setup—0% MLR and RTT results—showing a majority of MAVLink messages being responded to in under 17 ms while the maximum response time did not exceed 36 ms. In this case, the observed delay is introduced by a software protocol converter running on a single-board computer, responsible for bi-directional conversion of MAVLink messages between serial and IP transport.

### 4.1. USV Control/CBR Payload Traffic Link Sharing

Having established the optimistic case of the USV control loop having exclusive use of a highly reliable and communication link, we proceeded with the assessment of actual WLAN communication for this purpose. In the next series of experiments, we tested a MoIP communication loop over IEEE 802.11 radio link in three scenarios: (a) without any additional traffic, (b) sharing the link with CBR traffic while not exceeding the actual capacity of the link (50 Mbps—consistent with results of analysis in Section 3) and (c) sharing the link with CBR traffic, causing the link to be overloaded (100 Mbps).

**Figure 6:**
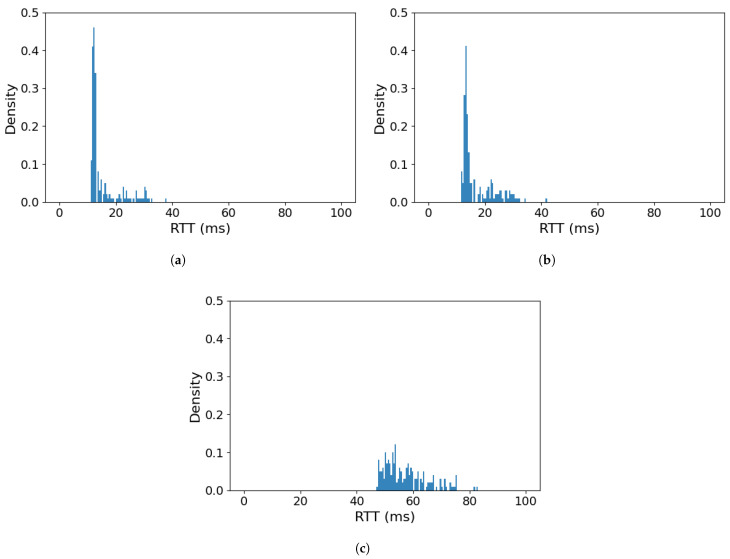
RTT value histogram for the MoIP control traffic (**a**) using a dedicated Wi-Fi link and for a control traffic sharing a Wi-Fi link with a CBR payload transmission: (**b**) Wi-Fi link not overloaded, (**c**) Wi-Fi link overloaded.

The results show that while the transmission of MoIP control traffic over a dedicated WLAN link results in a higher delay compared to an Ethernet link, it does not exceed 40 ms RTT, while most messages complete the round-trip within less than 20 ms (Figure 6a). The introduction of a CBR traffic stream transmitted from USV towards a receiver co-located with GCS and sharing the WLAN link with the control traffic (Figure 6b) does not change this results significantly, apart from causing a slightly higher percentage of messages to experience delay within 20–35 ms. This promising result remains valid as long as the aggregated throughput of control and payload traffic does not exceed the WLAN link capacity. If this capacity is exceeded (Figure 6c), we can expect control messages to be delayed by at least 47 ms and maximum delay to reach 82 ms.

Such RTT values indicate that while the impact of a payload traffic sharing a WLAN link with an USV control traffic is observable, it does not necessarily cause unacceptable degradation of communication quality. If the overall capacity of a link is not exceeded, the control loop delay is only slightly increased and acceptable for safe USV operation. However, the adverse effect of payload traffic, causing the capacity of a WLAN link to be exceeded, is much more pronounced and impacts delay of an USV control loop in a possibly unacceptable way, while in very good propagation conditions the experiment has been conducted in the values of RTT remain acceptable even for UAV control tasks, in real-world situations (where propagation conditions will be much worse), it can be expected that the absolute values of RTT will be much higher, while the relative impact of payload traffic will be at least as significant.

The above observations expose a serious problem, especially as we take into account a fact that the effective capacity of a WLAN link is not constant and depends on numerous hardware-specific characteristics (e.g., the sensitivity of a receiver, rate selection algorithm, etc.) and environment-specific factors, such as received signal strength and signal-to-interference and -noise ratio [36,37]. Moreover, the reaction of a Wi-Fi device to such changing conditions will differ between different COTS device models, due to the use of proprietary rate selection algorithms which are not configurable by the end user and have a great impact on the performance of a Wi-Fi device in changing propagation conditions [38]. Because many of the above environmental factors are subject to effectively unpredictable changes during USV operation in field conditions and because the use of different devices will result in employment of different rate selection mechanisms, we have to assume that it will be impossible to predict WLAN link capacity changes precisely enough to ensure that a link shared by USV control and payload traffic does not become overloaded.

### 4.2. Employing IEEE 802.11e Traffic Prioritization

Having reviewed possible approaches to the above problem, we decided to verify the applicability of traffic prioritization mechanisms introduced in the IEEE 802.11e [39] amendment to IEEE 802.11 standard as a potential solution. The mechanisms can be expected to be available in the majority of modern Commercial-Off-The-Shelf (COTS) Wi-Fi devices and allow network traffic to be divided into four classes, each assigned a different priority in the contention-based channel access utilized by Wi-Fi networks (Table 2).

The IEEE 802.11e prioritization mechanism works by differentiating parameters of the contention-based transmission medium access procedure. The value of Arbitration Interframe Space (AIFS) controls how long (in timeslots dependent on the type of a physical layer in use) a wireless device must detect a free medium before it can proceed with the next steps of the procedure. Contention window size parameters $CW_{min}$ and $CW_{max}$ (also defined in timeslots) place boundaries on a growth of a random backoff interval utilized if a device detects an already-occurring transmission while attempting to access the medium. The Transmission Opportunity (TXOP) parameter controls whether the device needs to perform a medium-access procedure for each IEEE 802.11 frame it wants to transmit (TXOP = 0) or if a successful medium-access procedure allows the device to transmit many frames in a time period specified by TXOP value (TXOP > 0).

As we are analyzing a case of a GCS-USV link which is expected to provide communication for clearly defined services (USV control loop and payload), in further analysis, we are going to omit the Background (BG) access class (intended for safe handling of unplanned traffic loads) as it is not applicable in this case. As in actual communication devices, IEEE 802.11e mechanisms (also called Wireless Multimedia Extensions, WMEs [40]) are most often enabled by default and configured to use the Best Effort (BE) class for all traffic, experiments presented in previous section, which used default configuration, effectively utilized the BE class for both USV control and payload traffic.

**Figure 7:**
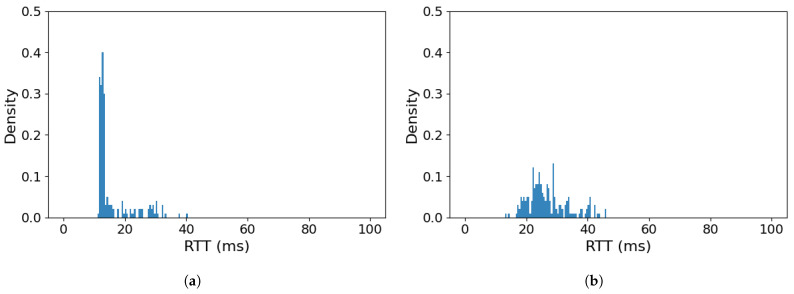
RTT value histogram for MoIP control traffic in VO priority class sharing a Wi-Fi link with a BE CBR payload transmission: (**a**) Wi-Fi link not overloaded, (**b**) Wi-Fi link overloaded.

Requirements of USV control traffic (high reliability and low delay) combined with low throughput make the use of a voice class (VO) a natural solution in this case—its parameters maximize probability of obtaining medium-access quickly (low AIFS, $CW_{min}$ and $CW_{max}$ values) in both low and high network load conditions, while a modest TXOP time is consistent with low throughput requirements. It can be expected to provide the control traffic with low latency access and ensure its clear priority over the payload traffic, which remains assigned to BE class—Figure 7 illustrates such a scenario.

As can be seen in Figure 7, the effect of employing IEEE 802.11e traffic prioritization is clearly observable in the case of the overloaded link (Figure 7b). Employing the VO traffic class for MoIP traffic allowed the delay to be contained within 45 ms, making the transmission delay range roughly similar to those of the dedicated and the shared/not-overloaded link cases; however, delay values are not concentrated as strongly in the lower end of the range. Such a result indicates that the employed method could indeed be highly effective in preventing the adverse effects of the unexpectedly changing data rate of a Wi-Fi link and the intermittent link overload conditions it is expected to cause. In the case of a shared Wi-Fi link in non-overload condition (Figure 7a), the use of VO traffic class for MoIP communication is not necessary, as the results obtained in this case are effectively the same as without prioritization (Figure 6a)—the maximum RTT it does not exceed 40 ms, with most messages being responded to within less than 20 ms.

The above laboratory results confirm the effectiveness of IEEE 802.11e traffic prioritization in ensuring that USV control traffic will be delivered between GCS and USV with a sufficient quality to satisfy the requirements of a real-time USV control, even in the presence of high-throughput payload traffic sharing the same WLAN link. Moreover, it remains possible even in the case when the link becomes overloaded, which solves the main problem that we expect to encounter in a field deployment of such a communication solution.

**Figure 8:**
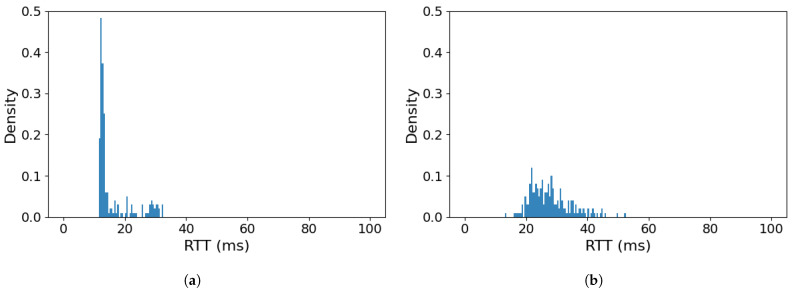
RTT value histogram for a MoIP control traffic in VO priority class sharing a Wi-Fi link with a CBR payload transmission in VI class: (**a**) Wi-Fi link not overloaded, (**b**) Wi-Fi link overloaded.

The previous experiment clearly illustrates that assigning the MoIP control traffic to a VO class can prove highly effective when it needs to compete with high-intensity, best-effort (BE) payload traffic for limited transmission resources. However, it should also be verified if this advantage will remain in effect when the competing payload traffic will be assigned to a high-priority class, such as video (VI). Such a situation can easily arise, because current implementation practices make Wi-Fi devices assign access classes based on Differentiated Services Code Point (DSCP) [41] values carried as a part of IP header, which are assigned by applications—an option which modern multimedia applications often employ. The VI class is capable of providing a considerable throughput (as its TXOP is 2 times as large, as in the case of the VO class) while still keeping the delay in medium access small, due to low AIFS $CW_{min}$ and $CW_{max}$ values. At the same time, values of $CW_{min}$ and $CW_{max}$ parameters are higher than in the case of the VO class, which protects the time-sensitive VO traffic from interference from VI traffic streams in case of a high network load (when backoff mechanisms are frequently employed). Figure 8 presents a situation where payload traffic utilizes VI class (in keeping with its medium latency/high throughput requirements), while control traffic is assigned to the VO class, as before.

The histograms of RTT values for MoIP control traffic assigned to VO class (Figure 8) indicate, that sharing a Wi-Fi link with high-intensity traffic assigned to the VI traffic class has approximately the same effect, as observed in the case of the BE class. In the case of a shared Wi-Fi link in non-overload conditions (Figure 8a), all MoIP RTT values are contained in 15–35 ms range, which is an even better result than when competing payload traffic used the BE class (Figure 7a). In the case of an overloaded link (Figure 8b), almost all RTT values of MoIP traffic remain concentrated in the same range as in the previous experiment (17–45 ms), with no values exceeding 53 ms. At first glance, the slightly better results in non-overload conditions may seem counter-intuitive, but we need to remember that VI traffic class (in contrast to BE class) allows a TXOP mechanism to be utilized, which enables multiple Wi-Fi frames to be transmitted following a single medium-access procedure. This minimizes the number of times such a contention-based procedure needs to be utilized, thus limiting its impact on the VO-class control traffic.

The above results clearly confirm the advantages of employing traffic prioritization over Wi-Fi links employed for a shared USV control and payload traffic—moreover, they confirm efficiency of IEEE 802.11e mechanisms in this role. Classifying the MAVLink over IP control traffic as voice traffic class minimizes the impact of even high-intensity, constant bitrate (inelastic) payload traffic sharing the same Wi-Fi link on crucial Quality of Service parameters of USV control communication. The results indicate that use of the IEEE 802.11e VO class can be sufficient to maintain necessary QoS for USV control traffic in both non-overload and overload conditions. Additionally, the experiments show that classification of a high-intensity payload traffic sharing the link as the prioritized VI class does not significantly impact the QoS parameters of a VO-classified USV control traffic.

## 5. Unmanned Surface Vehicle Field Experiments

The laboratory experiments allowed the impact of a high-intensity payload traffic on a MAVLink over IP USV control loop communication to be observed in a number of scenarios. The experiments also indicated the need to employ a traffic prioritization solution while sharing a Wi-Fi link in such a way and confirmed the effectiveness of employing IEEE 802.11e mechanisms for this purpose. However, while the laboratory environment made it possible for us to observe and analyze specific interactions, such results are not sufficient to estimate the effectiveness of the proposed solution in its intended deployment scenarios due to the potentially unpredictable impact of a real-world propagation environment. To address this problem, a set of field experiments have been planned and conducted based on the results of laboratory experiments.

**Figure 9:**
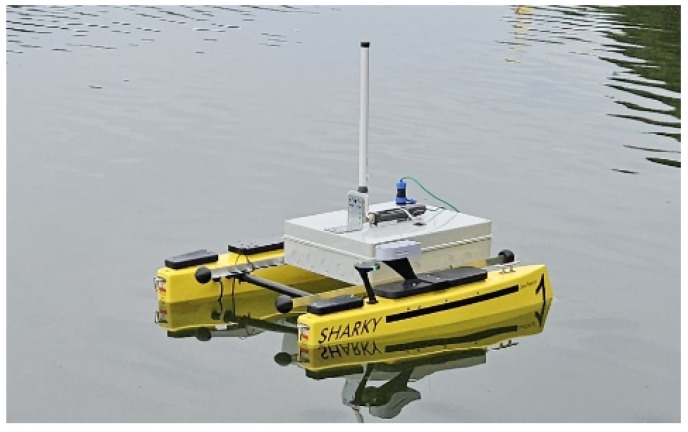
Geopixel Sharky USV with communication system-under-test installed.

For the field experiments, a Sharky (Geopixel, Poland, Figure 9) autonomous unmanned surface vehicle has been selected [42]. The USV is capable of autonomous operation based on predefined path and GPS location information, which allowed us to ensure that the USV movement path remained the same during successive experiments. The USV part of the testbed described in Section 4 (control system emulator, traffic generator, MAVLink converter, Gigabit Ethernet switch, Mikrotik Metal 52ac WLAN device with omnidirectional antenna) has been installed on the vehicle, separately from its actual control system. The remaining elements of the testbed have been co-located with GCS controlling the USV, also maintaining the separation. The USV omnidirectional antenna has been located at 0.5 m over the water level, while a GCS antenna has been installed 2 m over the water level and oriented in such way that USV constantly remained within its 3 dB sector (120°).

**Figure 10:**
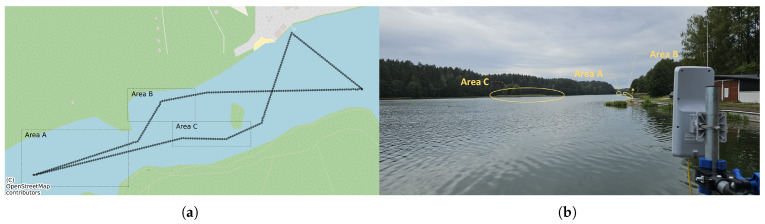
The USV deployment path (**a**) and a photo of deployment environment (**b**) with three areas of special interest.

To verify the operation of the communication system in scenarios expected during real USV deployments, the path that the USV followed at 1.5 m/s is presented in Figure 10a. Apart from requiring the communication system to operate under changing USV orientation relatively to GCS and wind direction (which can be significant due to different pitch and roll movements) and during slight and significant course changes, the selected path included three areas of special interest (Figure 10):

- Area A—located at an empirically obtained limit of a stable USV control loop communication distance possible using the communication system under test (about 750 m from GCS location),
- Area B—where Line of Sight (LoS) path between GCS and USV is obstructed by a densely overgrown lake shoreline,
- Area C—where LoS path between GCS and USV is obstructed by a moderately overgrown island.

The environment selected for field experiments lacked significant sources of outside radio interference, which has been confirmed by analyzing Signal-to-Noise Ratio (SNR) measurements gathered during experiments. In all cases, the SNR value changes remained consistent with changes in the signal strength due to propagation conditions.

Three experiments based on scenarios selected using laboratory results have been sequentially conducted over the same USV movement path described above:

- Experiment 1 (EXP-1)—WLAN link used exclusively for a MAVLink over IP control loop communication,
- Experiment 2 (EXP-2)—WLAN link shared between a MoIP control loop and an uplink 50 Mbps CBR transmission, both traffic flows using BE access class,
- Experiment 3 (EXP-3)—WLAN link shared between a MoIP control loop using VO access class and an uplink 50 Mbps CBR transmission using VI class.

**Figure 11:**
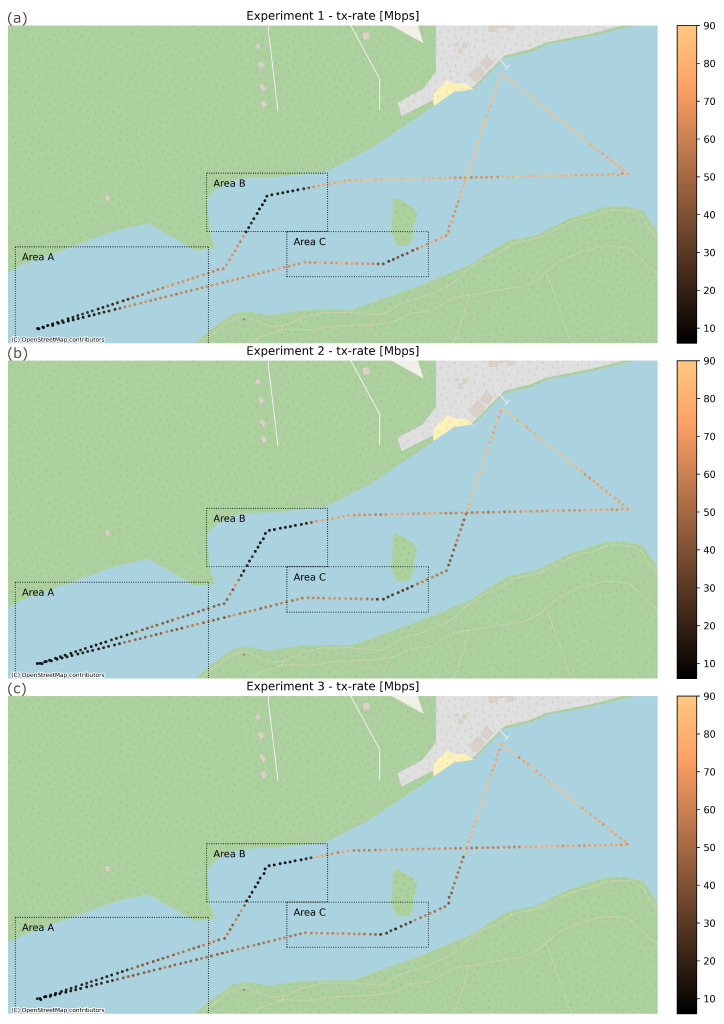
Wi-Fi uplink transmission rate during field USV experiments: (**a**) Experiment 1 (dedicated MoIP link), (**b**) Experiment 2 (shared MoIP/payload link), and (**c**) Experiment 3 (shared MoIP/payload link with traffic prioritization).

As can be seen in Figure 11, a degradation in propagation conditions (both caused by long communication range and LoS obstructions) results in a reduction in Wi-Fi link transmission rate between USV and GCS. Analyzing the results, it can be observed that LoS obstructions (Areas B and C) cause an immediate and substantial reduction in communication rate. Furthermore, transmission rate reduction due to range (Area A), while not as sudden as that caused by LoS loss, also happens quickly—in all experiments, the distance separating the first significant indications of transmission rate reduction due to range and the point where transmission were severely impaired did not exceed 75 m (about 10% of the maximum communication range).

**Figure 12:**
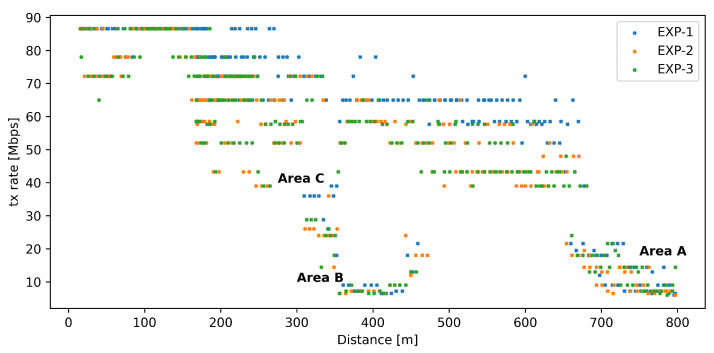
Wi-Fi transmission rate values recorded during Experiments 1–3 as a function of distance.

Recognizing the importance of Wi-Fi transmission rate selection algorithms, we have also analyzed Wi-Fi transmission rate values recorded during experiments, as a function of USV distance from the ground station. Figure 12 presents the results with additional markings to indicate groups of points corresponding to areas of special interest (introduced in Figure 10). The plot shows that in the case of the network under heavy load (Experiments 2 and 3), rate selection remains independent of the presence of our proposed mechanisms, as results are very similar in both cases. However, it is also visible that under minimal load (Experiment 1), the Wi-Fi transmission rate selected by rate selection mechanisms is generally higher. Because rate selection mechanisms are proprietary and specific details on their operation are generally not available, we can only estimate that the cause is the dependency of their operation on transmission error and/or retransmission counters.

The results described above indicate that while it is relatively easy to designate areas where a reduction in available communication resources is likely to occur, the reduction itself is a rapid process. On this basis, it can be expected that the impact of unforeseen propagation obstacles (for example, shadowing by other vehicles) will also be sudden.

**Figure 13:**
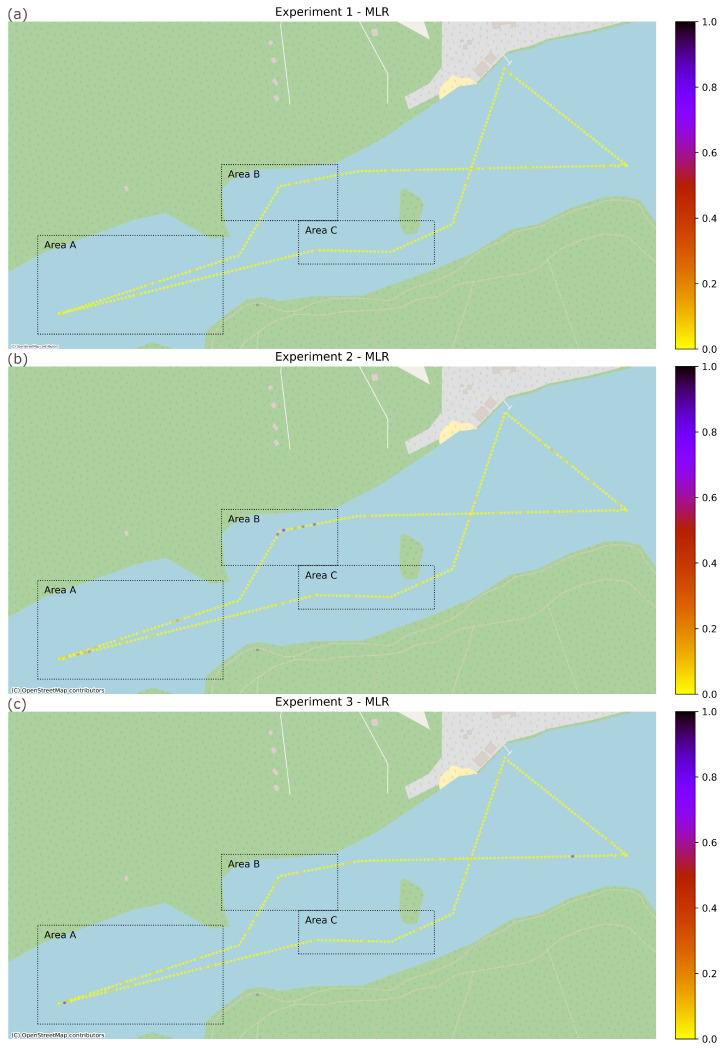
USV control loop MoIP Message Loss Ratio: (**a**) Experiment 1 (dedicated MoIP link), (**b**) Experiment 2 (shared MoIP/payload link), and (**c**) Experiment 3 (shared MoIP/payload link with traffic prioritization).

Keeping in mind this susceptibility of available transmission resources to propagation condition changes and the negative impact of overloading a wireless link on MAVLink control loop illustrated by laboratory experiments, the importance of verifying the values of Message Loss Ratio and Round Trip Time in real-world conditions is clear. In Figure 13, the MLR of the MAVLink control traffic is presented for all three experiments. The points have been plotted for intervals of 4 s and the value illustrates the percentage of MAVLink messages lost in a round-trip transmission during the 4 s period.

Furthermore, to facilitate a quantitative analysis of the experimental results, in Table 3, we present the statistical characteristics of MLR values for all the experiments. The table includes values of 99th percentile (P99, intended to illustrate worst case results) and mean and standard deviation (SD) for all areas of special interest defined in Figure 10 (where we expect challenging propagation conditions) and for the remaining area (where the propagation conditions are expected to be good).

As expected, based on laboratory experiment results, when the control traffic has exclusive of the Wi-Fi link (Experiment 1, Figure 13a), the losses are sporadic and affect isolated messages regardless of conditions (mean value of 0.01 with standard deviation smaller than 0.02 and 99th percentile below 0.07 in all areas). In the case of Experiment 2, where the control traffic is required to share the Wi-Fi link with a high-volume payload traffic (a 50 Mbps CBR uplink stream), degradation of propagation conditions results in periods of high message loss, exceeding 67% (P99 value of 0.672) when the link becomes overloaded due to decreased transmission rate (Figure 13b). This is a disturbing result, especially since continuous duration of these high loss periods often exceeds 5 s. In this situation, the practicality of employing a shared control-payload communication link can be questioned.

However, as illustrated by the results of Experiment 3 (Figure 13c), the proposed use of IEEE 802.11e prioritization mechanisms allows the losses to be significantly reduced. In the configuration chosen based on results of laboratory experiments, where control traffic utilizes VO class and payload traffic is transmitted using VI class, the loss periods become isolated (see Figure 11) and the P99 of MLR loss ratio calculated over 4 s intervals does not exceed 0.067. The gain is specially noticeable in challenging propagation conditions (Area B, no Line of Sight), where both the P99 and mean value of MLR has been reduced by the order of magnitude (P99 from 0.672 to 0.067 and mean from 0.065 to 0.006). Additionally, the value of SD is much lower (0.019 compared to previous 0.163), indicating much more stable communication conditions. The improvements resulting from adapting the proposed configuration bring communication quality to a level acceptable for a real-time USV control using MAVLink over IP protocol.

**Figure 14:**
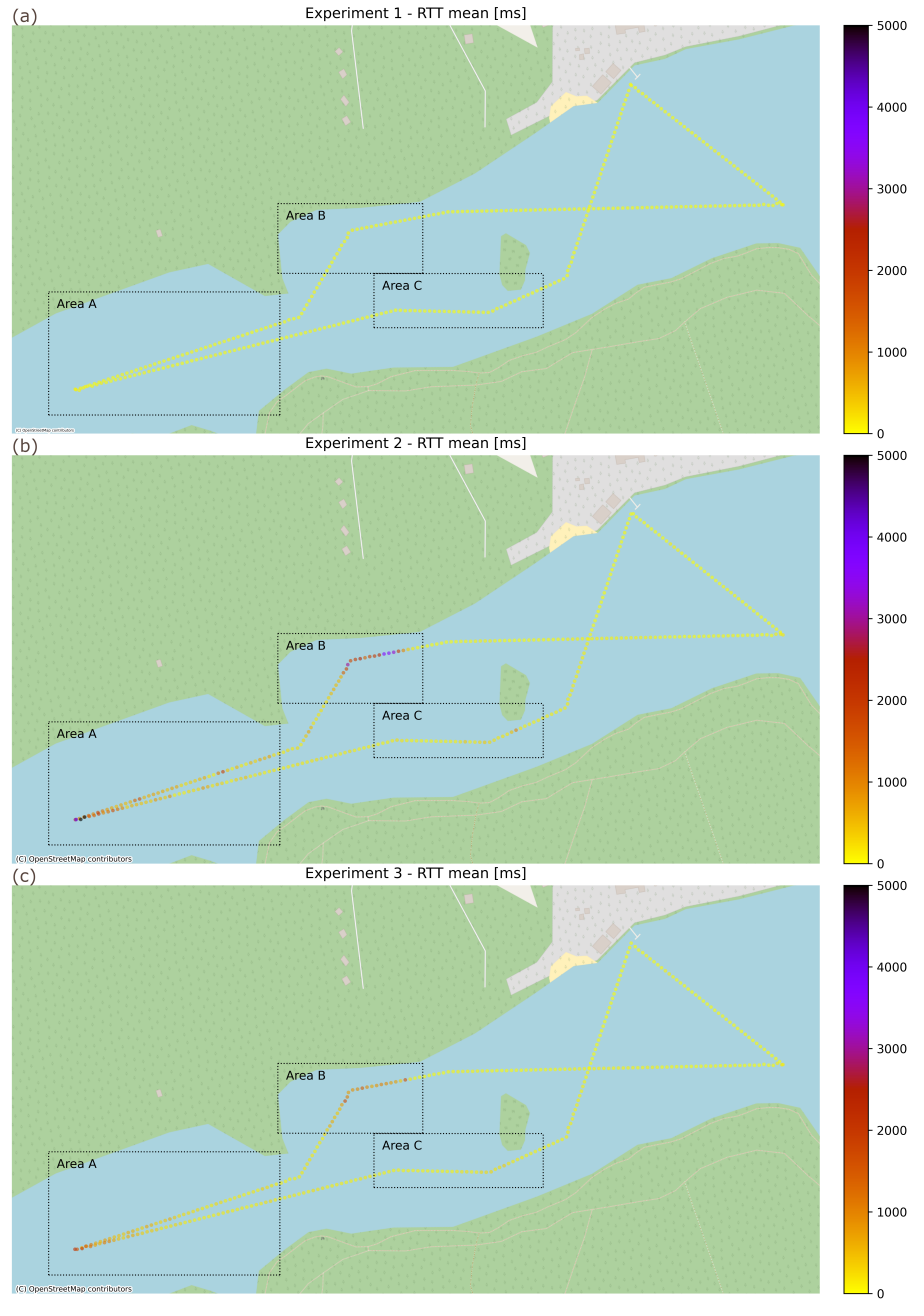
USV control loop MoIP message Round Trip Time: (**a**) Experiment 1 (dedicated MoIP link), (**b**) Experiment 2 (shared MoIP/payload link), (**c**) Experiment 3 (shared MoIP/payload link with traffic prioritization).

While message loss may be a serious problem, a method of employing MAVLink protocol for USV control takes this into account and provides a good level of resiliency to moderate MLR conditions by a continuous transmission of control signals using MAVLink messages sent repeatedly with a configurable interval for as long as a particular control input remains in effect. Therefore, excluding excessive loss conditions, this leaves a message transmission delay as a QoS characteristic of crucial importance for USV control, as it both directly influences a delay between a particular operator input and USV reaction and delays delivery of further messages due to buffering delay and head-of-line blocking effects. Values of a MAVLink message round-trip transmission delay for the discussed field scenario are presented on the map in Figure 14.

In the case of Experiment 1 (Figure 14a), it is evident that even in challenging conditions (Areas A, B and C) MAVLink message RTT remains very low, confirming the already-expected ability to provide a high-quality USV control loop using a dedicated Wi-Fi link. Unfortunately, it is no longer the case when the Wi-Fi link needs to be shared between control traffic and the high-intensity payload-related transmission (Experiment 2)—Figure 14b shows that in areas where propagation conditions were degraded and the resulting reduction in transmission rate caused the Wi-Fi link to be overloaded, transmission delay increased dramatically. Comparing this effect in Areas A and B, it can be seen that range-induced delay grows gradually, giving an operator a time to react, while LoS obstructions result in a very sudden delay increase. Moreover, a comparison of the results in Areas B and C indicates that the size and density of the obstruction is of significant consequence.

While the above results clearly indicate that sharing a Wi-Fi link between control and payload traffic in a real-world operational conditions (Experiment 2) can easily lead to link overload and serious degradation in the operator’s control capabilities, Experiment 3 confirms the (already-expected based on laboratory experiments) usefulness of IEEE 802.11e prioritization in such cases. As can be seen in Figure 14c, the negative impact of payload traffic is significantly reduced.

**Figure 15:**
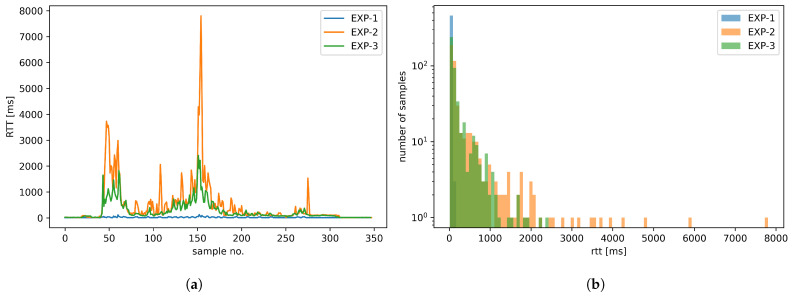
RTT values for a complete sequences of MAVLink control loop messages exchanged during field-tests (**a**) and corresponding histograms (**b**).

**Figure 16:**
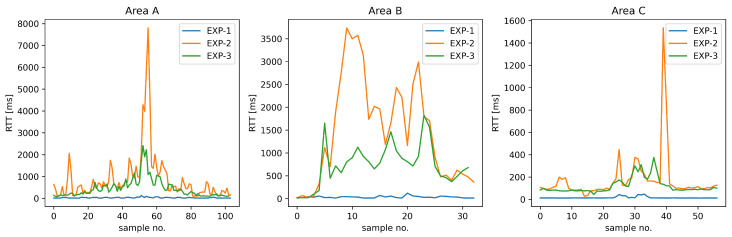
RTT values for sequences of MAVLink control loop messages in Areas A, B, and C.

To assess the performance USV control communications using Wi-Fi technology in field conditions, including the impact of the link overload and the ability to counteract it using traffic prioritization to regain the quality necessary for efficient USV remote operation, we have presented a sequential plot of RTT values for a complete path that USV followed during the above scenario (Figure 15a), supplemented by the corresponding histogram (Figure 15b). Additionally, in Table 4, we have provided statistical characteristics of RTT values for all the experiments, while RTT value sequences for the designated areas of special interest are presented in Figure 16.

When analyzing the RTT sequence plot and the histogram (Figure 15), the low latency of a dedicated Wi-Fi link, which is already observed on map plots, is clearly visible. Further analysis of statistical results presented in Table 4 confirms this assessment: even in poor propagation conditions (Area B), the P99 value does not exceed 102.5 ms, while the mean value is equal to 34 ms with SD of 21.7 ms. Such values indicate acceptable and relatively stable communication quality.

The negative impact of the high-intensity payload traffic on the control communication RTT when using the same (BE) traffic class (Experiment 2) is observable in all but the best communication conditions. In relatively good conditions, the effect takes the form of significant (500–1000 ms delay) and moderately frequent delay spikes (see Figure 15). Such characteristics and a P99 value of 429.3 ms still allows the USV control loop to operate in an acceptable manner. However, substantial LoS obstacles (Figure 16, Area B) result in a continuous RTT value increase of 1500–3700 ms, which exceeds the 2 s delay boundary of efficient USV control in diverse conditions (see Section 2). Statistical characteristics of P99 equal to 3683.8 ms, a mean value of 1472.3 ms with low stability indicated by SD value of 1121.9 ms further confirm negative assessment regarding control loop operation in these conditions. Moderate LoS obstacles (Figure 16, Area C) cause an observable RTT increase of 100–200 ms, with periodic spikes of about 200 ms and a single spike exceeding 1500 ms, illustrating a difficult-to-predict but significant impact of such events; while the mean RTT seems acceptable in this case (182.9 ms), the high SD of 233.3 ms indicates the low stability of its values. The results for the effective communication range boundary (Figure 16, Area A) show a gradual RTT growth with short-term 500–1500 ms spikes, culminating in a very fast (within 2 s) RTT increase to over 7 s. The RTT decreased in a similar fashion as the range decreased following the USV course change. A P99 value (5714.4 ms) higher than in case of Area B with a lower mean value is consistent with imminent communication loss due to range avoided by a quick course-reverse maneuver.

Assigning the control loop traffic to VO class and the high-intensity payload traffic to VI class (Experiment 3) provides a separate queue and medium-access control parameter values configured to minimize Wi-Fi access delay for delay-sensitive control communication. As a result, RTT values of MAVLink messages do not exceed a 2 s boundary anywhere along the recorded USV path. The only exception is the point marking a maximum range of reliable communication for this scenario, which has been selected due to this very reason—in this case, the P99 value reaches 2162.4 ms (see Table 4). In the remaining USV path, the P99 value does not exceed 1772.2 ms.

Comparison of RTT values (Figure 15) with Experiment 2 shows much smaller (200 ms maximum) RTT spikes during LoS operation within the communication range (outside of Areas A, B, and C). Statistical parameters for control traffic show a P99 value of 251.6 ms (previously 429.3 ms, 41% reduction) and a mean of 56.1 ms (previously 67.1 ms, a 17% reduction), indicating mitigation of momentary RTT spikes and confirming the good quality of service offered by the Wi-Fi communication system configured following the proposed guidelines.

In areas without LoS between USV and GCS (Areas B and C), the RTT values are significantly higher, but the P99 value still does not exceed 1772.2 ms (previously 3683.8 ms, a 52% reduction). Even at the extreme range (Area A), control and payload traffic link-sharing within the same class results in maximum RTT growing to over 7 s (Experiment 2, Figure 16, Area B), with the P99 value reaching 5714.4 ms, which may lead to a loss of operator control, in the case of the proposed configuration (Experiment 3), the RTT does not exceed 2200 ms (Figure 16, Area C) with P99 value of 2162.4 ms (62% reduction). This value is only slightly above the 2 s boundary, and this allows the operator to notice the necessity of taking a corrective action while still allowing them to do so effectively.

Such results illustrate that while sharing a communication link created using COTS Wi-Fi devices between control and payload traffic will lead to a degradation of quality of service for control traffic, the use of the proposed method of traffic prioritization is sufficient to keep its communication quality parameters within an acceptable range for USVs, even in adverse propagation conditions.

**Figure 17:**
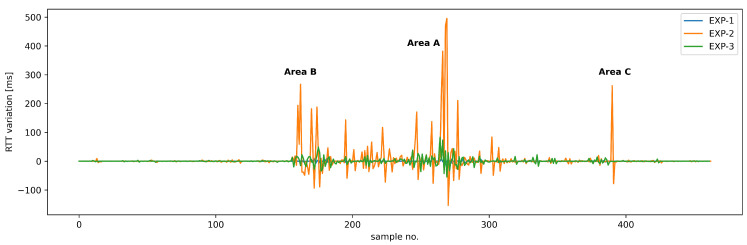
RTT variation values for complete measurement sequences of Experiments 1–3 with indicators for areas of special interests defined in Figure 10.

To further supplement the presented verification of the proposed link-sharing solution, we have also analyzed values of RTT variation calculated by using the approach employed in the IPDV method [43] for analysis of MAVLink RTT measurements (Figure 17). The results further illustrate the advantages of employing our proposed solution, as even in network-overload conditions, the values of RTT variation remain within levels acceptable for general USV control—they do not exceed 40 ms even in Areas A and B. Outside the areas of challenging propagation conditions, the RTT variation values for MAVLink control traffic remain comparable to values for similar traffic transmitted over a dedicated Wi-Fi Link (under 15 ms).

## 6. Conclusions

In this paper, we aimed to propose a method of employing the Wi-Fi technology as a universal communication solution for unmanned surface vehicles that is capable of not only maintaining a real-time control loop for an USV operator, but also carrying a substantial volume of payload-related traffic. Having confirmed the general applicability of Wi-Fi for USV control communication in our previous paper [10], this time we concentrated on the question of the above-mentioned link sharing.

For this purpose, a test system emulating a USV control loop using the MoIP protocol was designed and implemented. Then, a network architecture for a shared use of a wireless link by MoIP and a high-intensity payload traffic source (such as, for example, a high-resolution LiDAR) was prepared, implemented, and tested in laboratory conditions.

While the results confirmed the correct operation of the proposed link-sharing solution in normal network conditions, they also indicated the need for additional prioritization mechanisms to maintain sufficient quality of service for control traffic when the network becomes overloaded. As such a state can easily occur in Wi-Fi networks under field-conditions due to adaptive rate selection mechanisms responding to changing propagation environment, we proposed to employ IEEE 802.11e traffic prioritization functions and recommended a method of traffic class assignment. The proposed approach does not require any additional devices or technologies to be included, which facilitates its implementation in real-world USVs, as COTS communication solutions can be employed.

The proposed method of employing traffic prioritization mechanisms has been implemented and tested in both laboratory and field environments. The results confirm the effectiveness of our proposed USV link-sharing solution in a diverse real-world deployment conditions, including conditions of propagation degradation due to physical obstructions and extended communication range, where it allowed MLR P99 value reductions of 90% and 64%, respectively, while RTT P99 values were reduced by 52% and 62%.

The proposed solution and obtained experimentation results, confirming the applicability of properly deployed Wi-Fi technology as a universal communication solution for unmanned surface vehicles, can provide a significant advantage in the further development of USV communications, as WLAN technologies can easily be used to create complex communication systems providing extended coverage or improved reliability. However, we need to keep in mind that the presented experiments assume a lack of significant external interference from neighboring wireless systems or jamming sources. While it can be expected that the proposed method of deployment will provide some mitigation of the adverse results, the verification of their impact on USV communications will be pursued in our future paper.

## Figures and Tables

**Table 1:** Traffic model characteristics.

	Traffic Type	Message Size	Data Stream	Direction
MAVlink	CBR	112 bits	1200 bps	bidirectional
Payload service	CBR	1470 bytes	50 Mbps	USV to GCS

**Table 2:** IEEE 802.11e access classes.

Access Class	Medium-Access Characteristics	Access Parameters According to IEEE 802.11e [39]
Voice (VO)	Low latency, standard throughput	AIFS = 2 $CW_{min}$ = $\frac{aCW_{min}+1}{4}-1$ $CW_{max}$ = $\frac{aCW_{min}+1}{2}-1$ TXOP = 1.504 ms
Video (VI)	Reduced latency, high throughput	AIFS = 2 $CW_{min}$= $\frac{aCW_{min}+1}{2}-1$ $CW_{max}$ = $aCW_{min}$ TXOP = 3.008 ms
Best Effort (BE)	Standard priority of medium access	AIFS = 3 $CW_{min}$ = $aCW_{min}$ $CW_{max}$ = $aCW_{max}$ TXOP = 0
Background (BG)	Reduced priority of medium access	AIFS = 7 $CW_{min}$ = $aCW_{min}$ $CW_{max}$ = $aCW_{max}$ TXOP = 0

$aCW_{min}=15$ and $aCW_{max}=1023$ are values defined by the IEEE 802.11 standard.

**Table 3:** Statistical characteristics of MLR values obtained during field experiments.

**Experiment 1 (Dedicated MoIP Link)**				
	**Area A**	**Area B**	**Area C**	**Remaining Area**
P99	0.067	0.067	0.035	0.067
mean	0.003	0.006	0.001	0.003
std	0.015	0.019	0.009	0.013
**Experiment 2 (Shared MoIP/Payload Link)**				
	**Area A**	**Area B**	**Area C**	**Remaining Area**
P99	0.187	0.672	0.067	0.067
mean	0.016	0.065	0.004	0.004
SD	0.042	0.163	0.016	0.016
**Experiment 3 (Shared MoIP/Payload Link with Traffic Prioritization)**				
	**Area A**	**Area B**	**Area C**	**Remaining Area**
P99	0.067	0.067	0.067	0.067
mean	0.017	0.006	0.004	0.005
SD	0.093	0.019	0.016	0.036

**Table 4:** Statistical characteristics of RTT values obtained during field experiments.

**Experiment 1 (Dedicated MoIP Link)**				
	**Area A**	**Area B**	**Area C**	**Remaining Area**
P99	90.0 ms	102.5 ms	45.5 ms	45.5 ms
mean	27.8 ms	34.0 ms	16.8 ms	14.4 ms
SD	19.1 ms	21.7 ms	9.2 ms	6.7 ms
**Experiment 2 (Shared MoIP/Payload Link)**				
	**Area A**	**Area B**	**Area C**	**Remaining Area**
P99	5714.4 ms	3683.8 ms	1205.1 ms	429.3 ms
mean	794.5 ms	1472.3 ms	182.9 ms	67.1 ms
SD	1100.1 ms	1121.9 ms	233.3 ms	82.7 ms
**Experiment 3 (Shared MoIP/Payload Link with Traffic Prioritization)**				
	**Area A**	**Area B**	**Area C**	**Remaining Area**
P99	2162.4 ms	1772.2 ms	344.5 ms	251.6 ms
mean	408.8 ms	743.6 ms	124.3 ms	56.1 ms
SD	409.1 ms	449.5 ms	72.7 ms	53.1 ms

## Data Availability

The original contributions presented in this study are included in the article. Further inquiries can be directed to the corresponding author.

## Abbreviations

The following abbreviations are used in this manuscript:

UV	Unmanned vehicle
USV	Unmanned surface vehicle
WLAN	Wireless local area network
VLOS	Visual Line of Sight
MAVLink	Micro Air Vehicle Link
TDMA	Time Division Multiple Access
LTE	Long-Term Evolution
QoS	Quality of service
LiDAR	Light Detection and Ranging
RTT	Round Trip Time
MLR	Message Loss Ratio
RADAR	Radio Detection and Ranging
SONAR	Sound Navigation and Ranging
LWIR	Long-Wave Infrared
HDR	High Dynamic Range
HEVC	High-Efficiency Video Coding
CBR	Constant bitrate
VBR	Variable bitrate
IEEE	Institute of Electrical and Electronics Engineers
GCS	Ground control station
MoIP	MAVLink-over-IP
UDP	User Datagram Protocol
ISM	Industrial, Scientific, Medical
COTS	Commercial-Off-The-Shelf
CW	Contention Window
AIFS	Arbitration Interframe Space
TXOP	Transmission Opportunity
BG	Background
BE	Best Effort
VO	Voice
VI	Video
WME	Wireless Multimedia Extension
DSCP	Differentiated Services Code Point
GPS	Global Positioning System
P99	99th percentile
SD	Standard deviation
